# Effects of Meal Timing on Postprandial Glucose Metabolism and Blood Metabolites in Healthy Adults

**DOI:** 10.3390/nu10111763

**Published:** 2018-11-14

**Authors:** Masaki Takahashi, Mamiho Ozaki, Moon-Il Kang, Hiroyuki Sasaki, Mayuko Fukazawa, Tamao Iwakami, Pei Jean Lim, Hyeon-Ki Kim, Shinya Aoyama, Shigenobu Shibata

**Affiliations:** 1 Waseda Bioscience Research Institute in Singapore, Waseda University, Singapore 138667, Singapore; p.lim@kurenai.waseda.jp; 2 Graduate School of Advanced Science and Engineering, Waseda University, Tokyo 1628480, Japan; mo_u2@ruri.waseda.jp (M.O.); hiroyuki-sasaki@asagi.waseda.jp (H.S.); yu8m1omo5iwo6@suou.waseda.jp (M.F.); iwakami.tamao@gmail.com (T.I.); 3 Human Metabolome Technologies Inc., Tokyo 1040033, Japan; mikang@humanmetabolome.com; 4 AIST—National Institute of Advanced Industrial Science and Technology, Waseda University Computational Bio Big-Data Open Innovation Laboratory (CBBD-OIL), Tokyo 1690072, Japan; 5 Organization for University Research Initiatives, Waseda University, Tokyo 1628480, Japan; hk.kim@aoni.waseda.jp (H.-K.K.); saoyama@aoni.waseda.jp (S.A.); 6 Faculty of Science and Engineering, Waseda University, Tokyo 1628480, Japan; shibatas@waseda.jp

**Keywords:** incretin, metabolome, meal timing

## Abstract

We examined the effects of meal timing on postprandial glucose metabolism, including the incretin response and metabolites in healthy adults. Nineteen healthy young men completed two trials involving blood collection in a fasting state and at 30, 60 and 120 min after meal provision in a random order: (1) morning (~0900 h) and (2) evening (~1700 h). The blood metabolome of eight participants was analyzed using capillary electrophoresis-mass spectrometry. Postprandial glucose concentrations at 120 min (*p* = 0.030) and glucose-dependent insulinotropic polypeptide concentrations (*p* = 0.005) at 60 min in the evening trials were higher than those in the morning trials. The incremental area under the curve values of five glycolysis, tricarboxylic acid cycle and nucleotide-related metabolites and 18 amino acid-related metabolites were higher in the morning trials than those in the evening trials (*p* < 0.05). Partial least-squares analysis revealed that the total metabolic change was higher in the morning. Our study demonstrates that a meal in the evening exacerbates the state of postprandial hyperglycemia in healthy adults. In addition, this study provides insight into the difference of incretion and blood metabolites between breakfast and dinner, indicating that the total metabolic responses tends to be higher in the morning.

## 1. Introduction

Postprandial hyperglycemia is an independent risk factor for diabetes and cardiovascular disease [1,2,3]. Besides meal composition, meal timing has also been suggested to be an important factor regulating glucose tolerance, including insulin sensitivity, with elevated postprandial glucose levels reported to be higher in the evening than in the morning [4,5,6]. Other studies have indicated that late meals and an evening chronotype were associated with obesity, diabetes and metabolic syndrome [7,8,9]. However, the physiological mechanism that leads to these differences in postprandial glucose metabolism between morning and evening in humans remains unclear.One of the possible mechanisms resulting in such metabolic differences according to the time of day of eating could be related to the insulin secretion function, which has been reported to be significantly elevated in the morning compared to that in the evening and insulin sensitivity also decreases later in the day [10,11,12]. Some studies have shown that the insulin response to identical meals was higher in the morning than in the afternoon [10,13,14]. In addition, the levels of incretins such as glucose-dependent insulinotropic polypeptide (GIP) and glucagon-like peptide-1 (GLP-1), which act as stimulants of insulin secretion, also increase during the postprandial period [15,16]. Moreover, the serum concentrations of GIP and GLP-1 show a circadian rhythm with a peak in the afternoon [17,18,19]. The early release of GIP and GLP-1 was confirmed in another study showing their elevated levels in the morning compared to the afternoon after a standardized meal in healthy men [10]. Moreover, glucose metabolism including incretins was found to become worse in the afternoon in both normal glucose-tolerant subjects and in subjects with impaired fasting glucose and/or impaired glucose tolerance [14]. Collectively, these findings indicate that GIP and GLP-1 play an important role in mediating the differences in postprandial glucose metabolism between morning and evening.

Other possible mechanisms contributing to this difference could be related to differences in digestion, absorption and metabolism in the stomach and intestines that are modulated by circadian rhythms in mammals. Indeed, the levels of several transporters related to the absorption of glucose, peptides and lipids were found to be increased at night compared to those measured at daytime [20,21]. In particular, the genes encoding the transporters sodium/glucose cotransporter 1 (*Sglt1*), glucose transporter 2 (*Glut2*) and *Glut5* show clear circadian oscillations in their expression levels in rats [20]. Therefore, diurnal variation of these transporters would influence the metabolic response between morning and evening meals.

Recent advances in biological technology have enabled obtaining detailed information of the metabolome, which represents the entire profile of small-molecule metabolites that directly reflect the metabolic changes occurring in the body [22,23]. The measurement of specific metabolites in response to diet and exercise is also useful in the discovery of diagnostic and mechanistic biochemical biomarkers for assessing individual metabolic homeostasis [24,25]. In addition, some studies have shown that blood metabolomics could be used as an assessment of the human circadian rhythm [26,27]. In fact, the blood, urine and saliva human metabolomes were found to differ between morning and evening under both free-living and experimental conditions [22,23,28]. Moreover, changes in serum metabolites were demonstrated after an oral glucose challenge and a single meal [29,30,31,32]. A recent study further highlighted a relationship of dietary intake patterns and metabolic disease based on analysis of the gut microbiome and serum metabolome in lean and obese individuals [24]. Based on this background, we hypothesized that metabolomic profiles play a role in determining the postprandial glucose levels between morning and evening. However, to our knowledge, no study has directly compared the differences in acute metabolic responses, including changes in metabolomic profiles, that occur between the morning and evening meal in humans. Therefore, in the present study, we examined the effects of meal timing on postprandial glucose metabolism in healthy adults using a metabolomics approach. 

## 2. Materials and Methods

### 2.1. Participants

Nineteen healthy young men (aged 21–30 years) participated in this study after providing written informed consent. This study was conducted according to the guidelines laid down in the Declaration of Helsinki and was approved by the ethics committee of Waseda University (2015-252). The inclusion criteria were: (1) participants were not using glucose/insulin-lowering or related medication, (2) had not experienced a change in body weight of more than 5% within the past six months, (3) not heavy drinkers (men: <40 g/day), (4) not diagnosed with diabetes or dyslipidemia by a doctor and (5) not taking any anti-oxidant, anti-obesity, or anti-diabetes supplementation. All participants completed a questionnaire on physical activity, exercise, dietary intake, lifestyle habits and health status prior to the study. Although none of the participants was a trained athlete competing in any sporting events, some participants were recreationally active. The physical characteristics of the participants are summarized in Table 1.

Chronotype was assessed using the Horne-Ostberg Morningness-Eveningness Questionnaire (MEQ) [33], which consists of 19 questions related to preferred sleep time and daily performance (e.g., “what would be the best time to perform hard physical work?”). The scores ranged from 16 to 86. On the basis of their scores, the participants were divided into the following three chronotype groups: morningness (score 59–86), intermediate (score 42–58), or eveningness (score 16–41).

Eight of the healthy young men included in the study were selected for measurement of serum metabolites based on sample availability and cost. Given previous reports demonstrating an association of chronotype with the risk of diabetes [8,9], we selected two participants each from the morningness and eveningness groups and four participants from the intermediate group to equalize the chronotype distribution. The physical characteristics of these eight participants are presented in Table S1.

### 2.2. Main Trials

**Figure 1:**
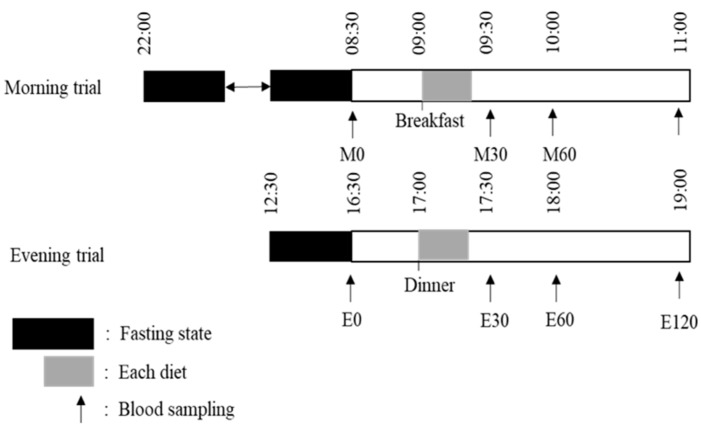
Protocols for the morning trial and evening trial.

A randomized crossover design was used. Each participant attended two laboratory-based tests in random order: (1) morning trial (~0900 h) and (2) evening trial (~1700 h) (Figure 1). The interval between trials was at least 1 week. For the morning trial, all participants were required to visit the laboratory at 0830 after a minimum 10 h overnight fast (no intake of food or drink, except water), whereas for the evening trial, all participants were required to visit the laboratory at 1630 after a 4-h fasting state (no intake of food or drink, except water). For both trials, after a 10–15 min rest, a fasting venous blood sample was collected by venipuncture while the participants were in a seated position. Further venous blood samples were collected at 30, 60 and 120 min after providing the test meal in each trial.

### 2.3. Test Meals

The test meals in the morning and evening trials were identical, consisting of rice, pork, onion, green pepper, asparagus, bok-choy, enoki, cod roe, egg, vermicelli, carrot, leek and yogurt. The meal was prescribed according to body mass, with an average of 60 kJ energy/kg body mass as supervised by a registered dietitian. The energy of the meal was distributed as follows: 15% from fat, 70% from carbohydrate and 15% from protein. A previous study indicated that this percentage of carbohydrate loading can potentially increase postprandial glucose in healthy adults [34]. All participants were asked to consume the test meal within 20 min. The time taken to consume the first test meal (i.e., at the morning trial) was recorded and replicated in the subsequent test meal (i.e., at the evening trial). None of the participants reported nausea or any gastrointestinal discomfort in either trial. The participants consumed water *ad libitum* during the first trial and the pattern and volume ingested were replicated in the subsequent trial.

### 2.4. Standardization of the Meal and Physical Activity

All participants were asked to follow the same meal plan, which we provided, before the intervention for each postprandial test (i.e., supper for the morning trial and lunch for the evening trial). All participants refrained from drinking alcohol 1 day before each postprandial test and were asked not to change their dietary pattern during the experimental period. In addition, all participants were asked to refrain from remaining inactive or participating in high-intensity physical activity on the day before each postprandial test and throughout the testing period.

### 2.5. Blood Collection and Analysis

For plasma glucose measurements, the venous blood samples were collected into tubes containing sodium fluoride-ethylenediaminetetraacetic acid. The samples were immediately centrifuged at 3000 rpm for 10 min at 4 °C and the serum was dispensed into plain microtubes and stored at −80 °C until the assay. For measurement of incretins such as GLP-1 and GIP, venous blood samples were collected in tubes containing a DPP-IV inhibitor and protease inhibitor cocktail (BD, Tokyo, Japan). To measure serum insulin, venous blood samples were collected in tubes containing clotting activators for isolation of serum. Samples were allowed to clot for 30 min at room temperature and then centrifuged and treated as described above. Enzymatic colorimetric assays were used to measure the plasma concentrations of glucose (GLU-HK (M); Shino-test Corporation, Kanagawa, Japan). Plasma concentrations of insulin (Mercodia Insulin ELISA; Mercodia AB, Uppsala, Sweden), total GIP (EMD Millipore ELISA; Merck Millipore, Merck Ltd., Danvers, MA, USA) and total GLP-1(EMD Millipore ELISA; Merck Millipore, Merck Ltd., Danvers, MA, USA) were measured by enzyme-linked immunosorbent assay. 

### 2.6. Blood Metabolome Analysis

Fifty microliters of serum was added to 450 µL of methanol containing internal standards (Human Metabolome Technologies, Inc., Tsuruoka, Japan) at 0 °C to inactivate enzymes. The extract solution was then thoroughly mixed with 500 µL of chloroform and 200 µL of Milli-Q water, followed by centrifugation at 2300× *g* and 4 °C for 5 min. The 350 µL of the upper aqueous layer was centrifugally filtered through a Millipore 5 kDa-cut-off filter to remove proteins. The filtrate was centrifugally concentrated and re-suspended in 50 µL of Milli-Q water for capillary electrophoresis-mass spectrometry analysis. Metabolome measurements were conducted by Human Metabolome Technologies Inc., Tsuruoka, Japan.

### 2.7. Assessment of Blood Parameters and Insulin Sensitivity

Changes in the concentrations of blood parameters (glucose, insulin, GIP, GLP-1 and metabolome data) were assessed by the incremental area under the curve (iAUC), which was calculated by the trapezoidal rule. The homeostatic model assessment of insulin resistance (HOMA-IR) level was calculated according to the formula: fasting insulin (μU/mL) × fasting glucose (mg/dL)/405 [35,36].

### 2.8. Statistical Analysis

Data were analyzed using Predictive Analytics Software, version 23.0 for Windows (SPSS Japan Inc., Tokyo, Japan). The Kolmogorov-Smirnov test was used to check the normality of the distribution of all blood parameters. Unpaired Student’s *t*-tests were used to assess differences of data at the fasting state between the morning and evening trials and to compare the iAUC values. Two-factor repeated-measures analysis of variance (ANOVA) was used to determine the effects of the trial (morning or evening) and postprandial interval (0–120 min) on the concentrations of blood markers (glucose, insulin, GIP, GLP-1). When significant differences were detected at the fasting state, multivariate analysis of variance (MANOVA) was used to consider the values in the fasting state as a covariate. When significant interaction effects were detected, we used the Bonferroni method for post-hoc comparisons. Based on the distribution of postprandial glucose values in our previous study, the sample size was calculated to be able to detect a large effect (Cohen’s *d* = 0.98) [16,37]. Power analysis demonstrated that a sample size of 11 would be required to have approximately 80% power to detect a large effect at a significance level of 0.05. All data are presented as means ± standard error of the mean. Statistical significance was set at *p* < 0.05.

For serum metabolome data, hierarchical cluster analysis (HCA) and partial least squares (PLS) analysis were performed to visualize the data of metabolites and to assess the total metabolic change between the morning and evening trials using the heatmap 2 function in the gplots R package and HMT’s proprietary R program, respectively. For comparison of individual metabolites, we used the log-transformed values for statistical analysis due to the right-skewed distribution according to a previous study [29].

## 3. Results

### 3.1. Fasting Physical Characteristics and Blood Glucose, Insulin, GIP and GLP-1 Levels

**Figure 2:**
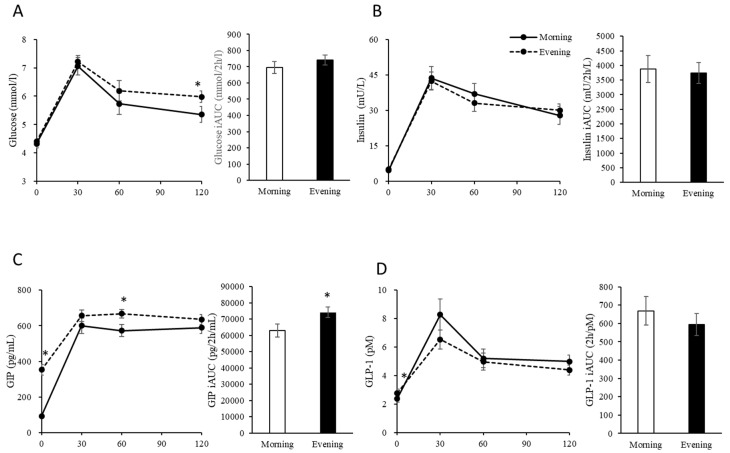
Concentrations and incremental area under the curve (iAUC) of plasma glucose (**A**), serum insulin (**B**), GIP (**C**) and GLP-1 (**D**) in the morning and evening trials. Values are means and standard errors represented by bidirectional bars. * Mean value was significantly different from that of the morning trials (*p* < 0.05).

There were no differences in the physical characteristics at the fasting state for all 19 participants between the morning and evening trials (Table 1). The chronotype distribution of the participants assessed by MEQ scores was highly skewed toward the intermediate category (*n* = 15), with two participants each in the morningness and eveningness groups. The concentrations of glucose, insulin and HOMA-IR (morning trials: 0.9 ± 0.1, evening trials: 1.0 ± 0.1) did not differ significantly between morning and evening trials. By contrast, the concentrations of GIP (*p* = 0.001) and GLP-1 (*p* = 0.041) at the fasting state were higher in the evening trials than those in the morning trials (Figure 2). 

### 3.2. Postprandial Blood Glucose, Insulin, GIP and GLP-1 Levels

Two-factor ANOVA revealed significant main effects of time (*p* = 0.001) and a trial × time interaction (*p* = 0.017) for plasma glucose concentrations of all 19 participants (Figure 2). Post-hoc tests showed that the concentrations of glucose in the evening trials at 120 min (*p* = 0.030) after meal provision were significantly higher than those in the morning trials. A significant main effect of time was detected for serum insulin concentrations (*p* = 0.001). The iAUC of blood glucose and insulin levels did not differ significantly between morning and evening trials.

For plasma GIP concentrations, MANOVA revealed significant main effects of trial (*p* = 0.001) and time (*p* = 0.001) and a trial × time interaction (*p* = 0.001) (Figure 2). Post-hoc tests showed that concentrations of GIP in the evening trials at 60 min (*p* = 0.005) after meal provision were significantly higher than those in the morning trials. Similarly, for plasma GLP-1 concentrations, MANOVA revealed a significant main effect of time (*p* = 0.001) and a trial × time interaction (*p* = 0.022) (Figure 2). The iAUC of GIP was higher in the evening trials than that of morning trials (*p* = 0.019). However, the iAUC of GLP-1 levels did not differ significantly between morning and evening trials.

### 3.3. HCA and PLS Analysis of Blood Metabolome Data

**Figure 3:**
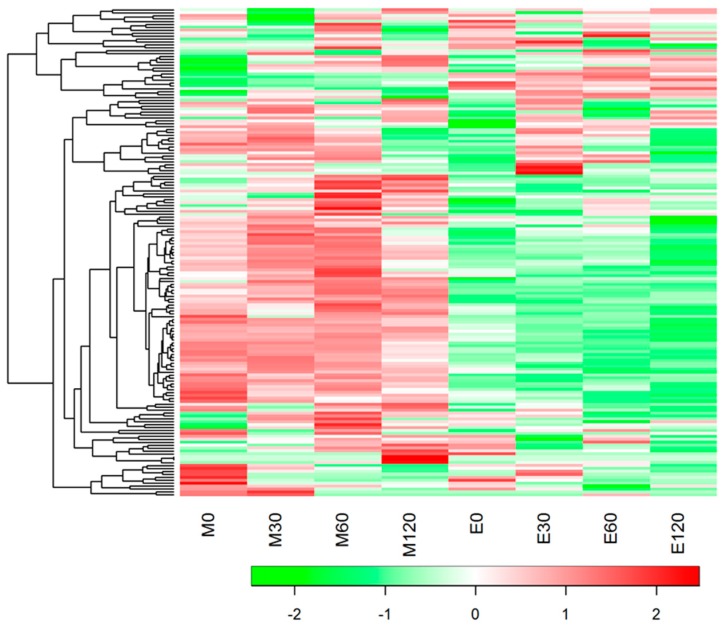
Heatmap of total metabolic profiles during the fasting state and postprandial state.

**Figure 4:**
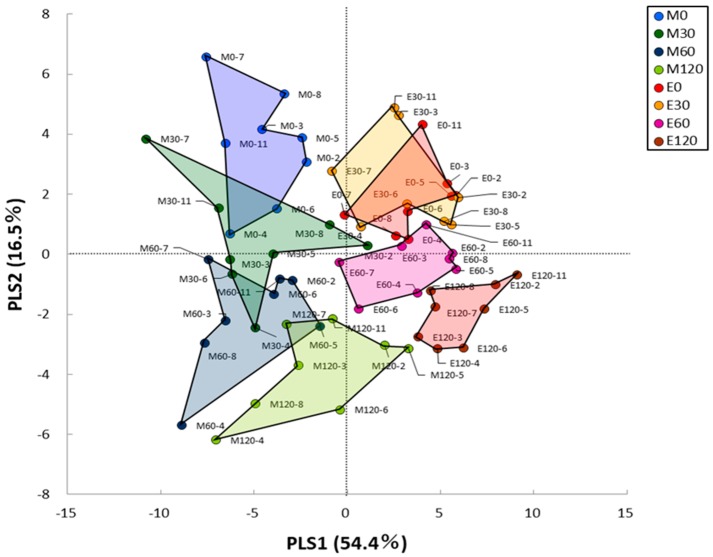
Partial least squares analysis of total metabolic changes from the fasting to postprandial state.

We measured serum metabolites by the capillary electrophoresis-mass spectrometry method for eight of the participants at the fasting and postprandial state. HCA separated all 164 metabolites detected into two main clusters (Figure 3). The list of all metabolites is provided in Supplemental Table S2. Cluster analysis revealed higher levels of metabolites at the fasting state in the evening trials (#1), at both the fasting and postprandial state in the evening trials (#2), at both the fasting and postprandial state in the morning trials (#3) and at the fasting state in the morning trials (#4) (Figure S1). The total metabolic changes between the morning and evening trials were identified by PLS analysis (Figure 4). The PLS model considering trial (morning and evening trials) as a covariate showed complete separation between trials (total amount of variance explained in the x matrix (cumulative) = 54.4%; total amount of variance explained in the y matrix (cumulative) = 16.5%). 

**Figure 5:**
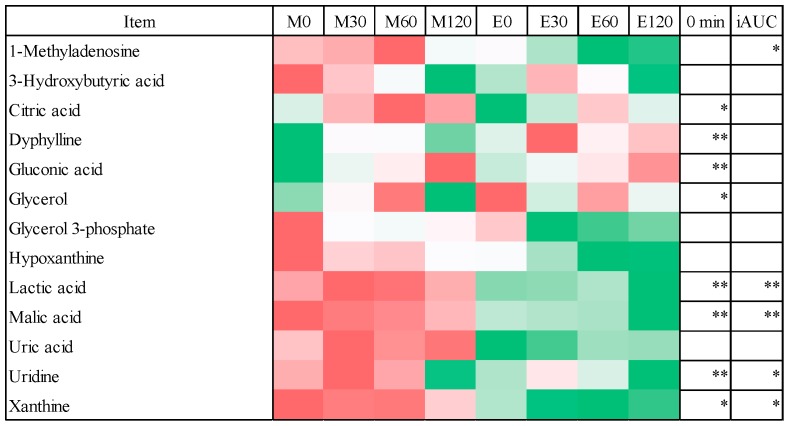
Heatmap and statistical analysis of glycolysis, TCA and nucleotide-related metabolites. * *p* < 0.05 vs. morning trials, ** *p* < 0.01 vs. morning trials.

**Figure 6:**
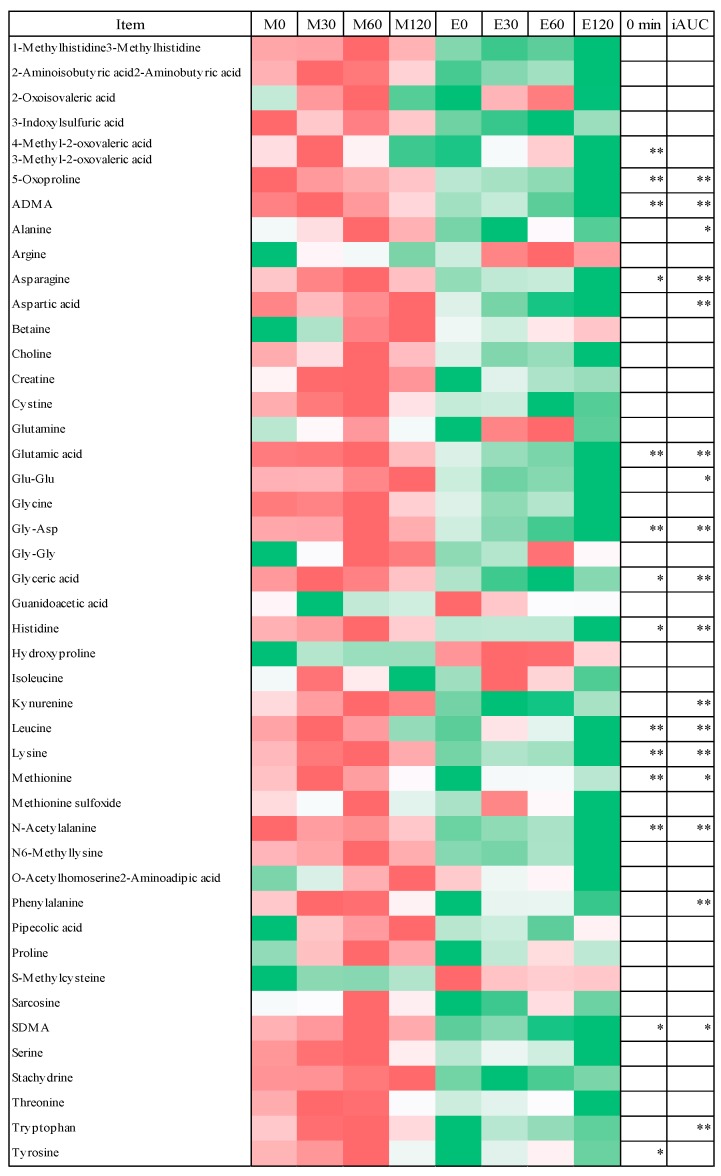
Heatmap and statistical analysis of amino acid related metabolites. * *p* < 0.05 vs. morning trials, ** *p* < 0.01 vs. morning trials.

To compare individual metabolites between the morning and evening trials, we evaluated the 83 metabolites detected at all time points (Figure S2), which were categorized as glycolysis, tricarboxylic acid (TCA) and nucleotide-related (13 metabolites); choline, lipids and related (16 metabolites); urea, polyamine and related (10 metabolites); amino acids and related (45 metabolites); or others (11 metabolites). Some metabolites were categorized into more than two groups. Since the goal of the present study was to focus on glucose metabolism, we compared only the glycolysis, TCA and nucleotide-related (13 metabolites) and the amino acid-related metabolites (45 metabolites) between the morning and evening trials (Figure 5 and Figure 6).

### 3.4. Fasting Blood Metabolome Data

In the fasting state, there were significant differences detected between the morning and evening trials in eight glycolysis, TCA and nucleotide-related metabolites (Figure 5) and in 14 amino acid-related metabolites (Figure 6). The levels of five of the glycolysis, TCA and nucleotide-related metabolites and all of the amino acid-related metabolites were higher in the morning trials than those in the evening trials (*p* < 0.05 or *p* < 0.01). Conversely, three glycolysis, TCA and nucleotide-related metabolite (dyphyline, gluconic acid and gluconic acid) was higher in the evening trial than in the morning trial.

### 3.5. Postprandial Blood Metabolome Data

The iAUC values of five glycolysis, TCA, nucleotide-related metabolites (Figure 5) and 18 amino acid-related metabolites (Figure 6) were higher in the morning trials than those in the evening trials (*p* < 0.05 or *p* < 0.01). 

## 4. Discussion

To our knowledge, this is the first study to examine the effect of meal timing on multiple human metabolites based on metabolomics profiling. The main finding is that the postprandial glucose level was elevated in the evening compared to that in the morning. By contrast, the postprandial incretin response and almost all of the blood metabolites detected were elevated in the morning compared to those detected in evening trials. These findings suggest that the timing of a meal should be considered when examining the overall effects of meal intake on metabolism and incretin. Moreover, metabolome data provide a useful resource for detecting changes in specific postprandial blood metabolites during morning and evening meals.

Under normal conditions such as during sleep at night and consuming a regular meal during the day, glucose tolerance tends to deteriorate from the morning to the evening in healthy adults [4,13]. However, some studies have indicated that the postprandial glucose level was more strongly elevated following the evening meal than after the morning meal [4,5,13]. Our results are consistent with these previous studies since the concentrations of glucose in the evening trials at 120 min after meal provision were significantly higher than those detected in the morning trials. These responses may be associated with the decrease of the insulin sensitivity in the evening and reduced early-phase insulin secretion caused by an insufficient B-cell response [10,13]. Previous studies have shown that the concentrations of insulin were elevated in the evening meal compared to those measured during the morning meal [4,5]. However, we did not find a significant difference in the insulin concentrations between morning and evening trials. These discrepancies between studies may be attributed to the characteristics of the participants included in our study compared to others. For example, only males were included in our study, whereas other studies included participants of both sexes. Therefore, the menstrual cycle phase and sex hormones may influence insulin sensitivity [38,39,40]. In addition, our study was conducted in a Japanese population, whereas previous studies were conducted in the USA and Europe [4,5,6]. Another study conducted with a Japanese population showed a lower rate of insulin secretion (first-phase insulin secretion) than that of a Caucasian population [41]. Moreover, the Japanese population has a higher prevalence of postprandial hyperglycemia than the Caucasian population [1,2], which is mostly due to diminished first-phase insulin secretion [41].

The physiological contributors to daily variations in insulin secretion, including higher morning beta-cell function and action in the pancreas, could reflect the circadian rhythms in the concentrations of cortisol and adrenocorticotropic hormone as well as in the meal-induced glucagon responses [42,43]. In addition, incretins such as GLP-1 and GIP act as stimulants for insulin secretion and are elevated during the postprandial period [15,16]. In the present study, the concentrations of GIP at the fasting state and at 60 min after meal provision in the evening trials were higher than those detected in the morning trials. In addition, the iAUC of GIP was significantly higher in the evening trials than that of morning trials. By contrast, some studies have found a higher iAUC for GIP and GLP-1 in the morning [10,14]. One possible factor responsible for these differences may be related to the characteristics of participants such as age, sex, race and body mass index as well as insulin function. Moreover, postprandial GIP and GLP-1 responses would be influenced by the contents of test meal such as total energy and energy balance. Thus, these differences may be explained some discrepancies of GIP and GLP-1 responses between our study and other previous studies. Incretins, also known as gastrointestinal hormones, are influenced by meal stimulation both directly and indirectly. In particular, GIP is secreted by K cells located on the upper part of the small intestine, which is more susceptible to the influence of food intake than GLP-1 that, is secreted by L cells. In fact, the change of the concentrations of GIP from the fasting state to the postprandial state at 30 min was higher in the morning trials than in the evening trials. One study demonstrated that the GIP and GLP-1 responses to identical meals were elevated after breakfast (~0900 h) than after supper (~1700 h) in healthy men [10]. Another study also demonstarated a high-carbohydrate and high-fat meal in the evening was associated with impaired glucose metabolism [14]. These results suggest that the function of insulin secretion, as assessed by incretin quantification, is reduced in the evening, leading to deterioration of postprandial glucose metabolism. Alternatively, the higher GIP responses in the morning could be related to the diurnal variation in the gastric emptying rate, which has been correlated with changes in incretin secretion [44]. These relationships need to be examined in more detail in future research.

Physiological processes that follow a circadian rhythm, such as digestion, absorption and metabolism in the stomach and intestines, influence the postprandial metabolism according to the time of day of eating. Previous studies have indicated that α-amylase, a glycolytic enzyme, has a circadian rhythm [45,46]. In addition, the levels of *Sglt1*, *Glut2* and *Glut5*, related to the absorption of glucose, peptides and lipids, were shown to increase at night compared to daytime. Similarly, GIP and GLP-1 concentrations have clear circadian oscillations, which peak in the afternoon [17,18]. Although there is no information available about the postprandial GIP and GLP-1 response in consideration of the same meal consumed at different times of day, one study demonstrated that the circadian phase and postprandial function of incretins such as GIP and GLP-1 in patients with diabetes were lower than those of healthy adults [18]. These previous findings support our speculation that a meal in the evening exacerbates postprandial hyperglycemia due to deteriorating incretin function, considering that the change of the concentrations of GIP from the fasting state to 30 min postprandial was lower in the evening than in the morning trials.

Some studies reported the changes in serum metabolites after an oral glucose challenge and a single meal [29,30,31,32,47]. Glucose load and meal intake elevate insulin secretion from the pancreas, which promotes glucose utilization by glucose oxidation and glycogenesis in the peripheral tissues such as the liver [30]. In turn, these responses suppress gluconeogenesis and glycogenolysis in the liver. Although the metabolic changes during the postprandial state are complex, they can now be investigated using metabolomics techniques by providing detailed information on different metabolic processes related to the entire metabolic status. Our metabolomics analysis revealed key differences of metabolites related to glycolysis, the TCA cycle, nucleotides and amino acids between the morning and evening trials. The metabolites related to glycolysis, the TCA cycle and nucleotides, such as citric acid, lactic acid and malic acid, were elevated at the fasting state in morning trials. Moreover, the iAUC values of methyladenosine, lactic acid, malic acid, uridine and xanthine were higher in the morning trials than in the evening trials. With respect to amino acid-related metabolites, aspartic acid, glutamic acid, glyceric acid, histidine, leucine, lysine, methionine and tyrosine were all found to be elevated in the fasting state at the morning trials. In addition, 16 amino acid-related metabolites, including arginine, asparagine, glutamic acid, histidine, leucine, lysine and tryptophan, were higher in the morning trials than in the evening trials. These findings suggest that postprandial metabolic changes between morning and evening meals are completely distinct.

A previous study showed that 15–20% of all identified metabolites in the plasma, saliva and urine were under circadian control, independent of sleep or meal intake [23,26,48,49]. Thus, several metabolites of the blood differ between morning and evening under free-living conditions. A more recent study evaluated the relationship between the gut microbiome and serum metabolome in lean and obese individuals; interestingly, they found that the disrupted circadian rhythm due to dietary intake patterns was correlated with metabolic disease [24]. However, the effects of an acute meal at different times on the serum metabolome have not been clarified. Indeed, we detected significant differences in several metabolites at the fasting state between morning and evening, most of which were higher in the morning. Although we assessed the postprandial metabolites based on the iAUC value, these differences may have nevertheless influenced the postprandial metabolic responses in both trials. However, the results of PLS analysis revealed that metabolic changes in all metabolites were higher in the morning trials than in the evening trials. In addition, the PLS analysis at the individual level showed high individual variation but similar trends of metabolic changes on average (Figure S3). Taken together, our data indicate that the declines of glucose metabolism and insulin function in the evening meal are associated with decreases of the glycolysis, TCA and nucleotide and amino acid metabolic pathways.

The present study has some limitations. First, the fasting period was shorter in the evening trial than in the morning trial so as to best mimic the daily lifestyle of most individuals. In general, the fasting period before breakfast is the longest in the human diet. Thus, the metabolic conditions of the participants in the present study were different from the general condition. Second, we could not evaluate some of the glycolysis and TCA cycle-related metabolites such as pyruvic acid, isocitric acid, succinic acid and fumaric acid at all time points. Therefore, additional research will be required to determine the changes in metabolites that could not be detected in the present study. In addition, participants with diabetes or hyperlipidemia were excluded from this study to focus on the general pattern in a healthy state. In the near future, it will be important to compare the metabolomic profiles between patients and healthy adults, such as with respect to the influence of diabetes and other metabolic disorders, with a focus on meal timing. Previous studies have indicated that the evening meal was correlated with the development of obesity, diabetes and metabolic syndrome [7,8,9]. Therefore, our results should be useful for designing future experiments focused on elucidating the mechanism underlying the metabolic difference of diet timing and searching for new risk factors and diagnostic biomarkers of diabetes using metabolomics analysis.

## 5. Conclusions

Our data demonstrate that a meal in the evening exacerbates the state of postprandial hyperglycemia in healthy adult men. In addition, most of the postprandial blood metabolites related to glycolysis, the TCA cycle and amino acids were elevated in the morning.

## Figures and Tables

**Table 1:** Physical characteristics and Home Ostberg Morningness Eveningness Questionnaire (MEQ) score of all participants in the morning and evening trials.

	Morning Trial	Evening Trial
	(*n* = 19)	(*n* = 19)
Age (years)	23.3 ± 0.6	23.1 ± 0.6
Height (m)	1.7 ± 0.1	1.7 ± 0.1
Body mass (kg)	64.6 ± 1.6	65.0 ± 2.1
BMI	22.0 ± 0.6	22.1 ± 0.5
Systolic blood pressure (mmHg)	120 ± 3	118 ± 3
Diastolic blood pressure (mmHg)	72 ± 2	71 ± 2
MEQ score	48 ± 2	

All data are presented as means ± SE.

## Supplementary Materials

The following materials are available online at http://www.mdpi.com/2072-6643/10/11/1763/s1, Figure S1: Characteristics of the heatmap of total metabolic profiles during the fasting state and postprandial state, Figure S2: Heatmap of detectable compounds extracted at all time points (84/167*), Figure S3: Partial least squares analysis of individual metabolic changes from the fasting to postprandial state, Table S1: Physical characteristics of the participants included in the serum metabolites analysis (*n* = 8), Table S2: The list of all metabolites shown in Figure 3.
