# Supplementary material for: Effects of Meal Timing on Postprandial Glucose Metabolism and Blood Metabolites in Healthy Adults
Source: Nutrients. 2018 Nov 14;10(11):1763. doi: 10.3390/nu10111763 (PMC6266071; doi:10.3390/nu10111763)
Supplement: Supplementary file 1 [file nutrients-10-01763-s001.zip › Supplemental files (Masaki Takahashi)/Supplemental Figures (Masaki Takahashi).pdf]

Figure S1

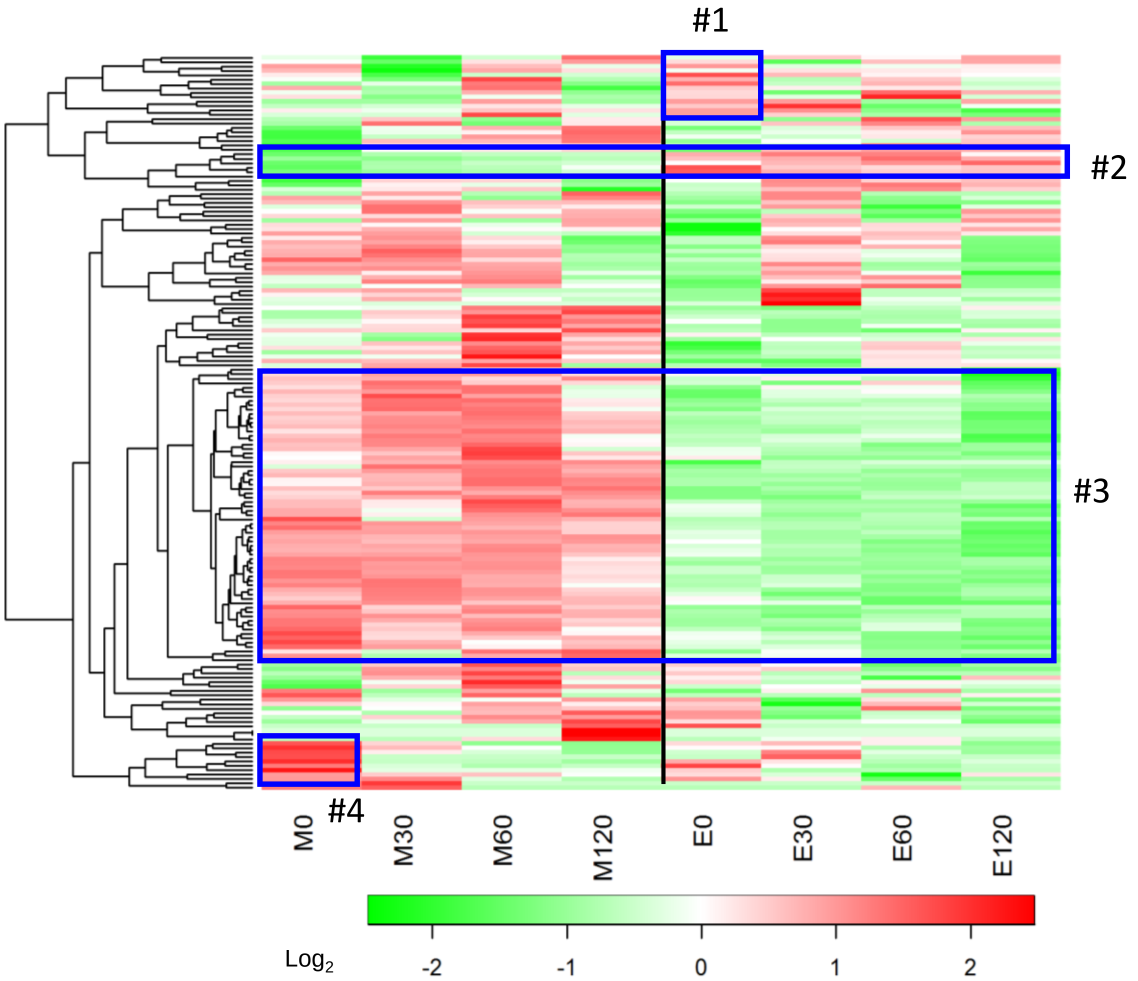

Figure S1 – Characteristics of heatmap of total metabolic profiles during fasting state and postprandial state.

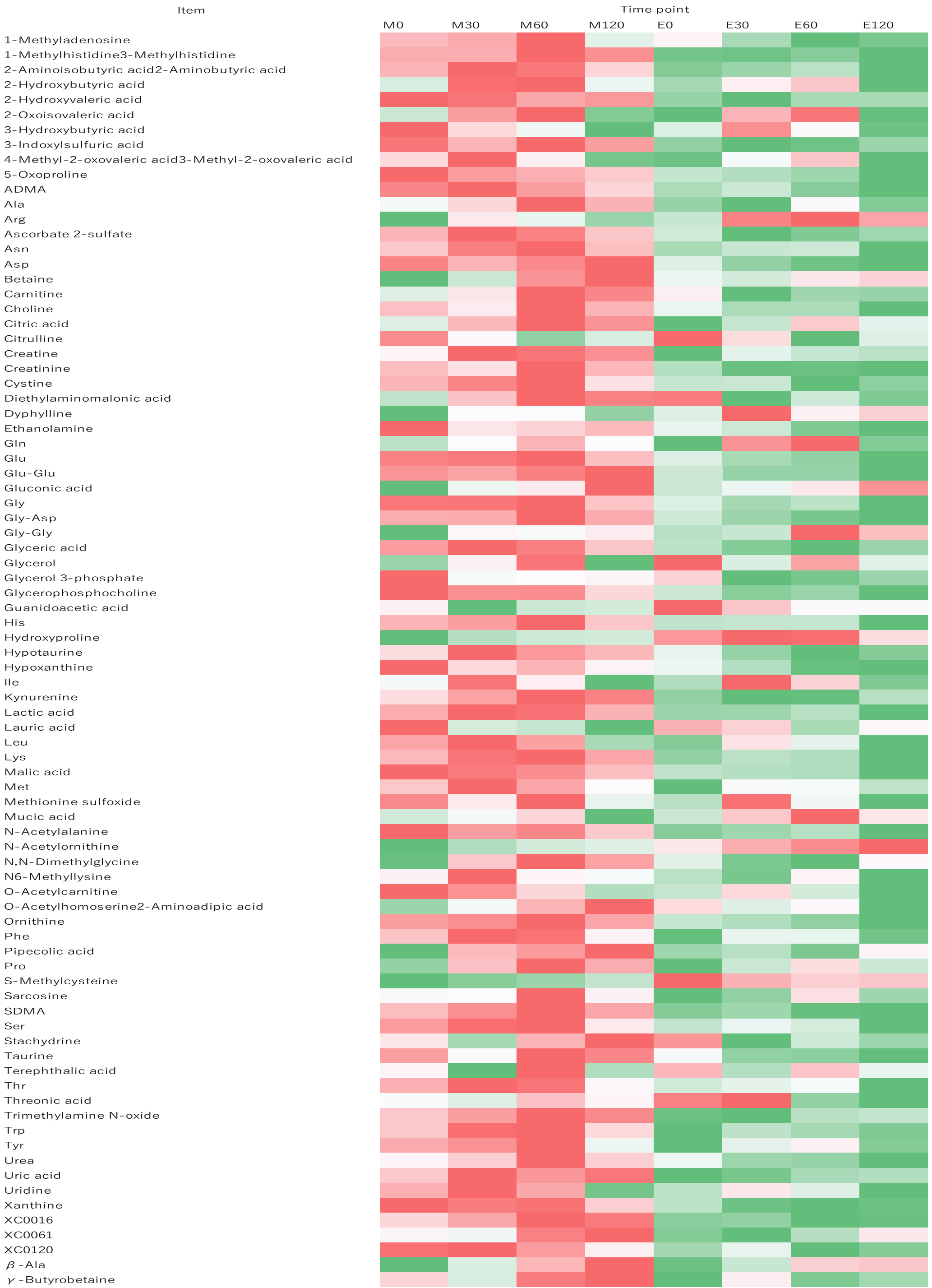

Figure S2 – Heatmap of extraction of detectable compounds at all time points (84/167*).

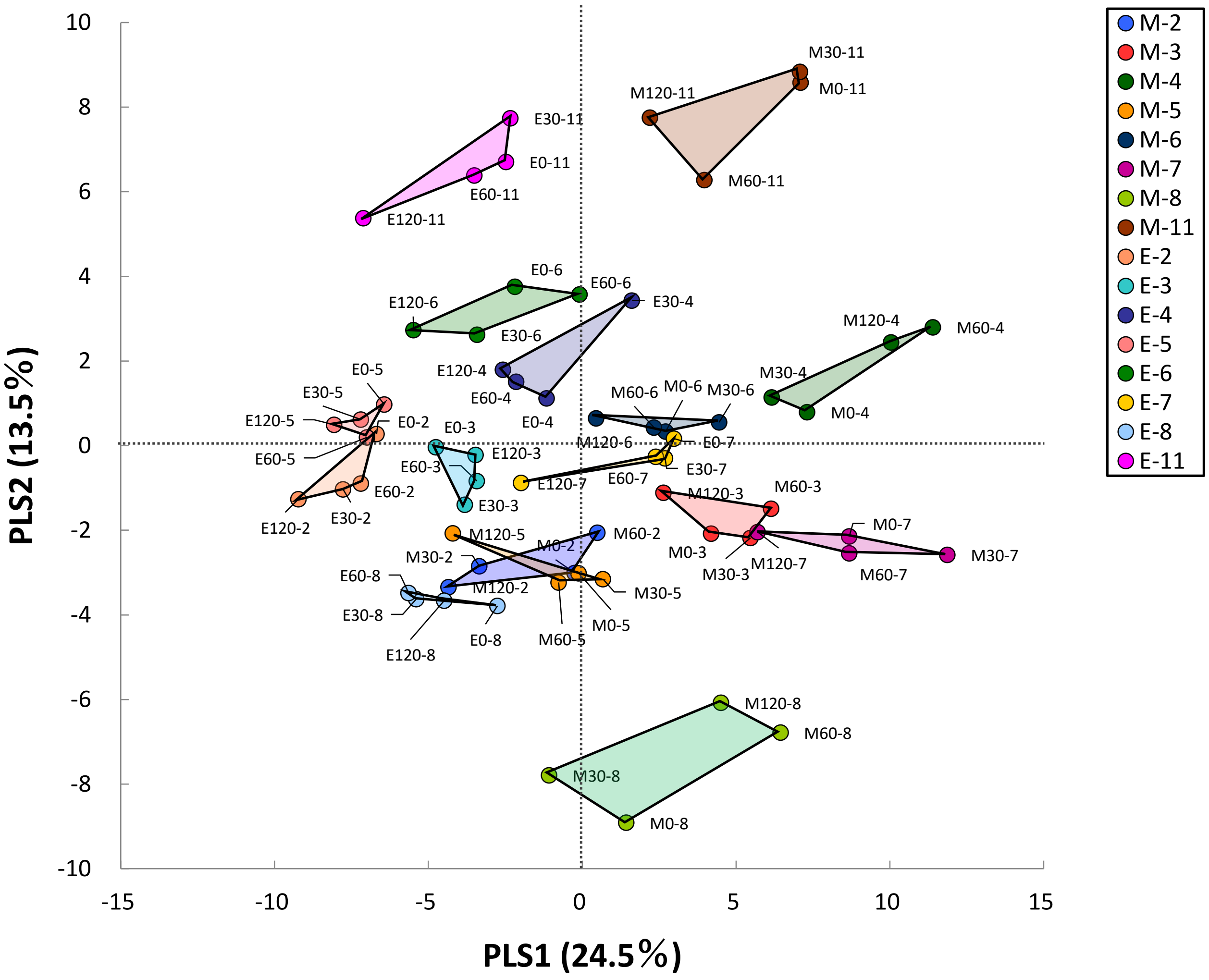

Figure S3 – Partial least squares analysis of individual metabolic changes from fasting to postprandial state.
