# Supplementary material for: Effects of Meal Timing on Postprandial Glucose Metabolism and Blood Metabolites in Healthy Adults
Source: Nutrients. 2018 Nov 14;10(11):1763. doi: 10.3390/nu10111763 (PMC6266071; doi:10.3390/nu10111763)
Supplement: Supplementary file 1 [file nutrients-10-01763-s001.zip › Supplemental files (Masaki Takahashi)/Supplemental Tables (Masaki Takahashi).pdf]

	Morning trial (n = 8)	Evening trial (n = 8)
Age (years)	23.4 ± 0.8	23.4 ± 0.8
Height (m)	1.7 ± 0.02	1.7 ± 0.02
Body mass (kg)	65.5 ± 4.3	66.0 ± 4.3
BMI	21.7 ± 1.1	21.9 ± 1.1
Systolic blood pressure (mmHg)	119.1 ± 2.9	119.1 ± 2.9
Diastolic blood pressure (mmHg)	70.3 ± 3.1	70.3 ± 3.1

Table S1. Physical characteristics of the participants who evaluated serum metabolites (n=8).
All data are presented as means ± SE.

Metabolities
Serotonin
Isobutyrylcarnitine
Urocanic acid
cis-4-Hydroxyproline
Guanidoacetic acid
Terephthalic acid
Glycerol
p-Hydroxybenzoic acid
XC0029
Indole-3-acetic acid
Isovaleric acid
Valeric acid
Cysteine glutathione disulfide
Threonic acid
Pelargonic acid
Theobromine
Homocitrulline
β-Ala
Gluconic acid
Betaine

Gluconolactone
Gly-Gly
Alliin
Hydroxyproline
Cystathionine
N-Acetylornithine
Trigonelline
S-Methylcysteine
Cyclohexylamine
Dyphylline
Arg
Mucic acid
Taurocholic acid
Perillic acid
N-Acetylputrescine
His-Glu
N^{8}-Acetylspermidine
7-Methylguanine
Caffeine
XC0132
N^{6}-Acetyllysine
2-Hydroxy-4-methylvaleric acid
Ile
Hexanoic acid
4-Methyl-2-oxovaleric acid
3-Methyl-2-oxovaleric acid
Uridine
Leu
O-Acetylcarnitine
Triethanolamine
N-Acetylglycine
Methionine sulfoxide
2-Oxoisovaleric acid
2-Hydroxybutyric acid
Gln

Glycocholic acid
Decanoic acid
Octanoic acid
Ascorbate 2-glucoside
XC0061
Pipecolic acid
N,*N*-Dimethylglycine
Carnitine
Ala
5-Methoxyindoleacetic acid
N^{5}-Ethylglutamine
Diethanolamine
Citric acid
cis-Aconitic acid
Pro
Sarcosine
Isovalerylcarnitine
N^{2}-Phenylacetylglutamine
XC0065
Nicotinamide
Butyrylcarnitine
N^{6}-Methyllysine
Val
Tyr
Met
Trp
2-Aminoisobutyric acid
2-Aminobutyric acid
Phe
His
Asn
Lys
Lactic acid
Aminoacetone
Thr

Ser
Cystine
1-Methyladenosine
Urea
N-Acetylgalactosamine
N-Acetylmannosamine
N-Acetylglucosamine
Isocitric acid
Creatine
SDMA
1-Methylhistidine
3-Methylhistidine
XC0016
Kynurenine
Homovanillic acid
Uric acid
Trimethylamine *N*-oxide
Creatinine
Choline
Taurine
Imidazolelactic acid
Succinic acid
Malic acid
5-Oxoproline
Gly
Glu-Glu
Asp
Ornithine
Gly-Asp
Glu
Paraxanthine
1-Methylnicotinamide
Xanthine
Glycerophosphocholine
Hippuric acid

ADMA
XC0120
Glyceric acid
Ascorbate 2-sulfate
Hypotaurine
Quinic acid
Pyruvic acid
3-Indoxylsulfuric acid
2-Hydroxyvaleric acid
N-Acetylalanine
2-Oxoglutaric acid
2-Hydroxyisobutyric acid
Hypoxanthine
Ethanolamine
Octanoylcarnitine
Guanidinosuccinic acid
γ-Butyrobetaine
Isethionic acid
Glucuronic acid
Galacturonic acid
N-Methylproline
Argininosuccinic acid
Ergothioneine
3-Aminopropionitrile
Acetohydroxamic acid
5-Hydroxylysine
Thr-Asp
Glutathione (GSSG)_divalent
Piperidine
3-Methoxytyrosine
N-Methylnorsalsolinol
Stachydrine
Diethylaminomalonic acid
O-Acetylhomoserine
2-Aminoadipic acid

Isopropanolamine
N-Methylglutamic acid
2-Hydroxyglutaric acid
5-Methylcytosine
Myristoleic acid
3-Phenylpropionic acid
3-Hydroxybutyric acid
10-Hydroxydecanoic acid
Lauric acid
Citrulline
Glycerol 3-phosphate
1-Methyl-4-imidazoleacetic acid
1-Methyl-2-pyrrolidone
N-Acetylglucosylamine
Fumaric acid

Table S2 The list of all metabolites of Figure 3
